# Supplementary material for: Highly Efficient Liquid-Phase Exfoliation of Layered Perovskite-like Titanates HLnTiO4 and H2Ln2Ti3O10 (Ln = La, Nd) into Nanosheets
Source: Nanomaterials (Basel). 2023 Nov 29;13(23):3052. doi: 10.3390/nano13233052 (PMC10708289; doi:10.3390/nano13233052)
Supplement: Supplementary file 1 [file nanomaterials-13-03052-s001.zip › nanomaterials-2738382-supplementary.pdf]

Supplementary Materials

Highly Efficient Liquid-Phase Exfoliation of Layered Perovskite-like Titanates $HLnTiO_4$ and $H_2Ln_2Ti_3O_{10}$ (Ln = La, Nd) into Nanosheets

Sergei A. Kurnosenko, Iana A. Minich, Oleg I. Silyukov * and Irina A. Zvereva

Department of Chemical Thermodynamics and Kinetics, Institute of Chemistry, Saint Petersburg State University, 199034 Saint Petersburg, Russia; st040572@student.spbu.ru (S.A.K.); yana.minich@spbu.ru (I.A.M.); irina.zvereva@spbu.ru (I.A.Z.)

* Correspondence: oleg.silyukov@spbu.ru

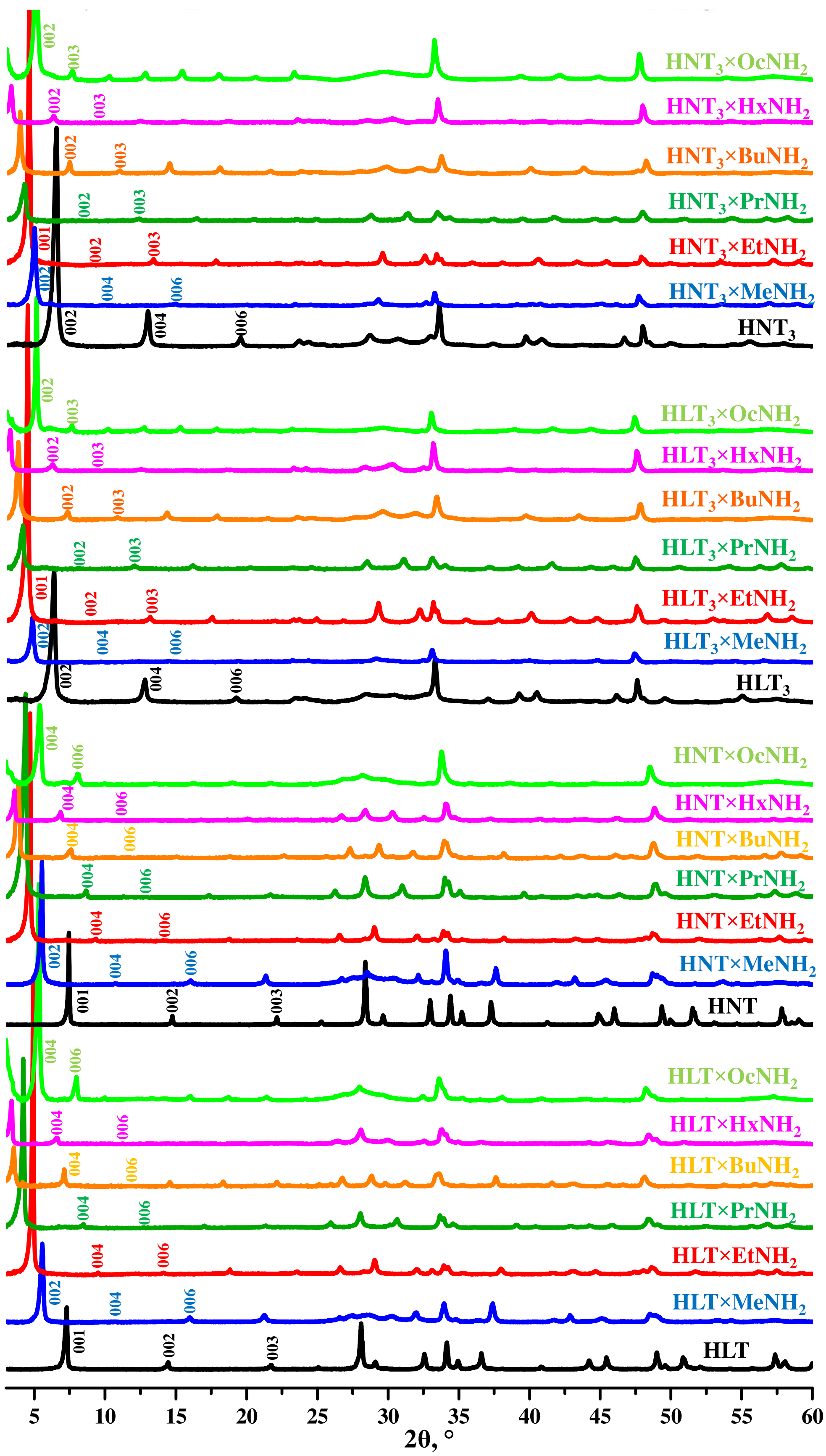

Figure S1. Powder XRD patterns of the protonated titanates and their *n*-alkylamine derivatives.

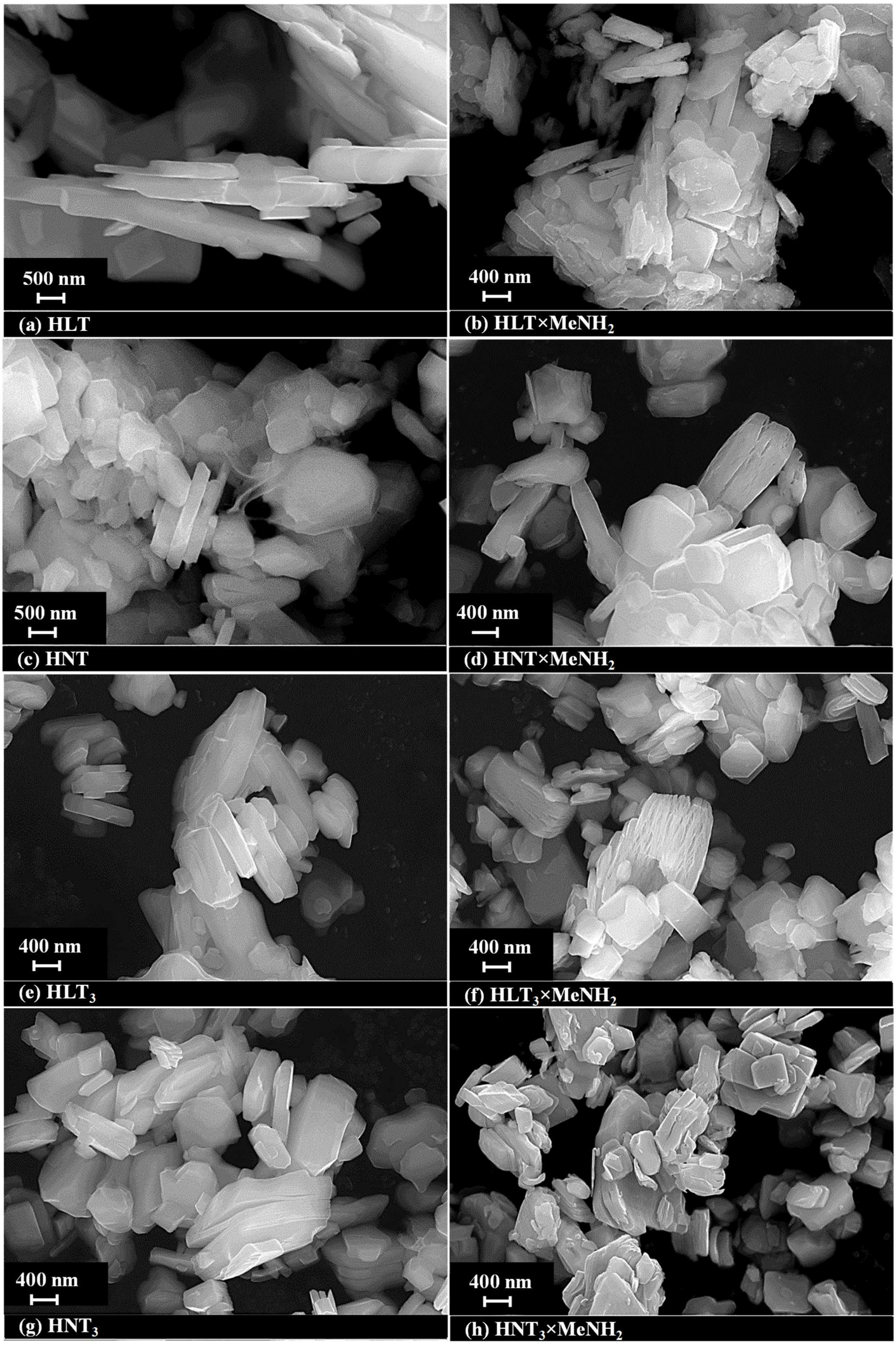

Figure S2. SEM images of the protonated and methylamine-intercalated titanates: HLT (a, b), HNT (c, d), HLT_3 (e, f) and HNT_3 (g, h).

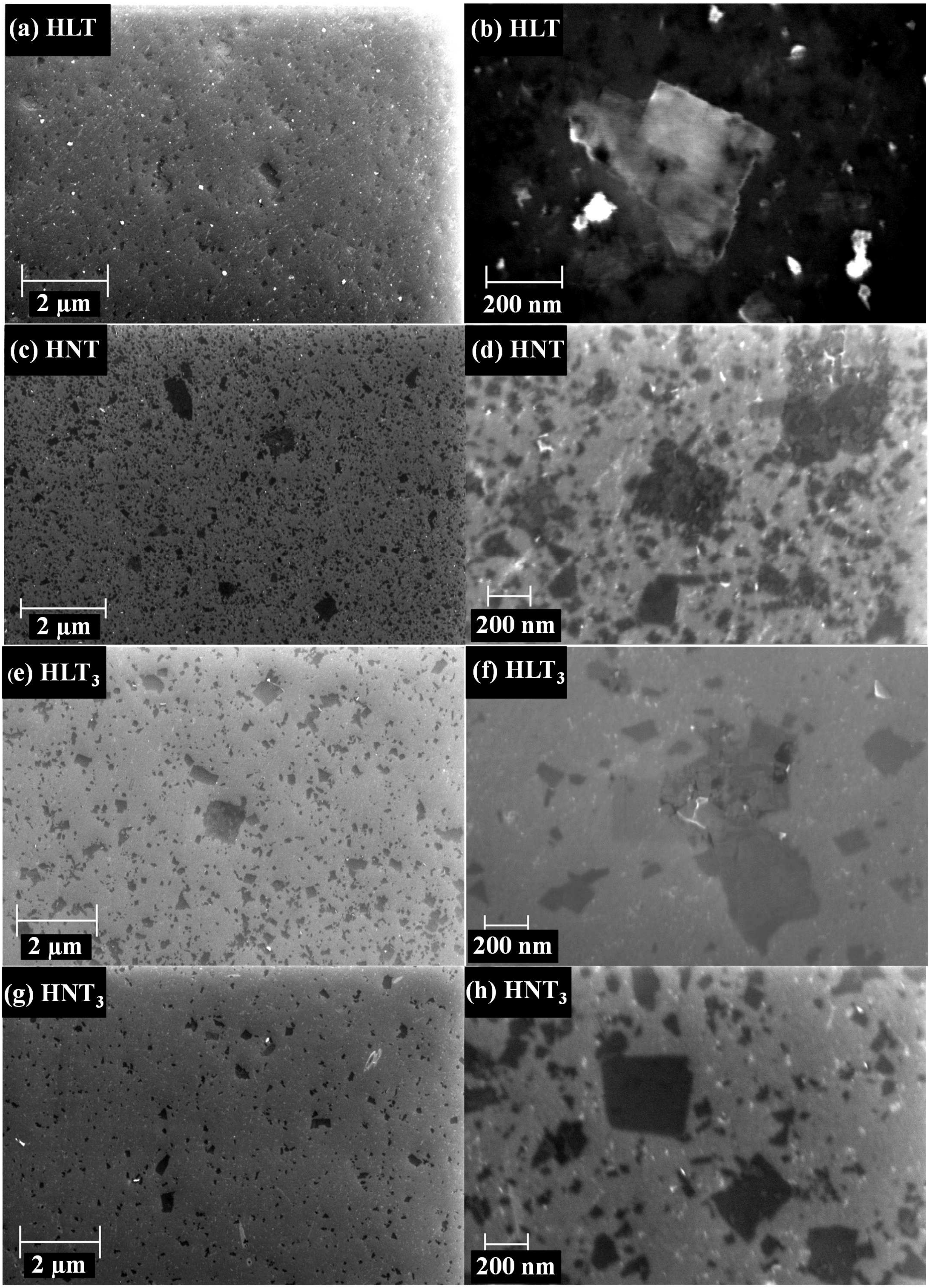

Figure S3. SEM images of HLT (a, b), HNT (c, d), HLT_3 (e, f) and HNT_3 (g, h) nanosheets on silicon substrates.

Table S1. Structure parameters and composition of initial compounds. Tetragonal lattice parameters, interlayer distances *d* as well as organic (x) and water (y) content per formula unit of the precursors for exfoliation. Compositions of the *n*-alkylamine derivatives are presented as $HLnTiO_4 \cdot xRNH_2 \cdot yH_2O$ and $H_2Ln_2Ti_3O_{10} \cdot xRNH_2 \cdot yH_2O$.

**Sample**	***a*=*b*, Å**	***c*, Å**	***d*, Å**	**x**	**y**	**Sample**	***a*=*b*, Å**	***c*, Å**	***d*, Å**	**x**	**y**
HLT	3.71	12.2	12.2	–	0.05	HLT_3	3.79	27.2	13.6	–	0.15
$\times MeNH_2$	3.77	33.3	16.7	0.35	0.45	$\times MeNH_2$	3.83	36.7	18.4	0.70	0.30
$\times EtNH_2$	3.75	37.7	18.9	0.35	0.50	$\times EtNH_2$	3.82	20.2	20.2	0.70	0.35
$\times PrNH_2$	3.76	41.2	20.6	0.35	0.30	$\times PrNH_2$	3.82	21.7	21.7	0.65	0.20
$\times BuNH_2$	3.76	47.8	23.9	0.40	0.25	$\times BuNH_2$	3.81	24.7	24.7	0.70	0.35
$\times HxNH_2$	3.76	54.9	27.5	0.35	0.45	$\times HxNH_2$	3.87	29.4	29.4	0.80	0.10
$\times OcNH_2$	3.78	66.3	33.2	0.45	0.40	$\times OcNH_2$	3.85	34.2	34.2	0.80	0.10
HNT	3.68	12.1	12.1	–	0.10	HNT_3	3.78	27.2	13.6	–	0.15
$\times MeNH_2$	3.74	33.1	16.6	0.35	0.40	$\times MeNH_2$	3.81	35.4	17.7	0.70	0.30
$\times EtNH_2$	3.74	37.6	18.8	0.45	0.20	$\times EtNH_2$	3.80	19.9	19.9	0.70	0.35
$\times PrNH_2$	3.74	40.4	20.2	0.45	0.40	$\times PrNH_2$	3.78	21.5	21.5	0.70	0.20
$\times BuNH_2$	3.74	47.7	23.9	0.45	0.30	$\times BuNH_2$	3.78	24.4	24.4	0.75	0.35
$\times HxNH_2$	3.72	54.0	27.0	0.45	0.35	$\times HxNH_2$	3.80	29.2	29.2	0.90	0.10
$\times OcNH_2$	3.81	65.4	32.7	0.45	0.40	$\times OcNH_2$	3.80	33.4	33.4	0.85	0.15

Table S2. Nanosheet concentrations and exfoliation yields achieved under various conditions. Designations: m – precursor weight, P – sonication power, t_1 – single sonication duration, t_2 – stirring duration, F – separation factor, c – nanosheet concentration, η – nanosheet yield.

**Variable parameter**	**Precursor**	**m, mg**	**TBAOH, M**	**Sonication**		**t_2, d**	**F**	**HLT**		**HNT**		**HLT_3**		**HNT_3**	
				**P, %**	**t_1, min**			**c, mg/L**	**η, %**	**c, mg/L**	**η, %**	**c, mg/L**	**η, %**	**c, mg/L**	**η, %**
**Physical exfoliation**															
–	Protonated	30	0	100	5	1	1000	1.5	0.15	1.7	0.17	1.6	0.16	1.8	0.18
	$\times MeNH_2$							3.7	0.37	6.3	0.63	3.8	0.38	6.1	0.61
**Chemical exfoliation**															
–	Protonated	30	0.004	–	–	1	1000	2.3	0.23	2.7	0.27	2.4	0.24	2.6	0.26
	$\times MeNH_2$							5.0	0.50	17	1.7	10	1.0	13	1.3
**Physical-chemical exfoliation**															
Chain length of *n*-alkylamine	Protonated	30	0.004	50	5	1	1000	4.1	0.41	4.7	0.47	2.6	0.26	3.2	0.32
	$\times MeNH_2$							46	4.6	530	54	100	10	330	33
	$\times EtNH_2$							50	5.0	750	75	170	17	460	46
	$\times PrNH_2$							48	4.8	510	51	140	14	79	7.9
	$\times BuNH_2$							13	1.3	6.5	0.65	4.5	0.45	8.6	0.86
	$\times HxNH_2$							11	1.1	5.3	0.53	4.1	0.41	5.4	0.54
	$\times OcNH_2$							3.6	0.36	4.9	0.49	3.7	0.37	4.0	0.40
Precursor weight	$\times EtNH_2$	90	0.012	50	5	1	1000	115	3.8	1500	50	340	11	960	32
		150	0.02					160	3.2	2100	42	480	9.6	1200	24
TBAOH concentration	$\times EtNH_2$	30	0.002	50	5	1	1000	21	2.1	290	29	85	8.5	260	26
			0.008					35	3.5	610	61	150	15	390	39
Sonication power	$\times EtNH_2$	30	0.004	25	5	1	1000	27	2.7	390	39	99	9.9	270	27
				100				52	5.2	740	74	180	18	450	45
Sonication duration	$\times EtNH_2$	30	0.004	50	1	1	1000	39	3.9	620	62	140	14	390	39
					10			53	5.3	770	77	180	18	480	48
Stirring duration	$\times EtNH_2$	30	0.004	50	5	7	1000	250	25	930	93	740	74	890	89
						21		270	27	950	95	810	81	920	92
Separation factor	$\times EtNH_2$	30	0.004	50	5	1	100	230	23	850	85	700	70	910	91
							500	95	9.5	770	77	310	31	720	72
